# Prediction of Drug Permeability Using *In Vitro* Blood–Brain Barrier Models with Human Induced Pluripotent Stem Cell-Derived Brain Microvascular Endothelial Cells

**DOI:** 10.1089/biores.2019.0026

**Published:** 2019-11-14

**Authors:** Makiko Ohshima, Shota Kamei, Hideo Fushimi, Shinji Mima, Tadanori Yamada, Takeshi Yamamoto

**Affiliations:** ^1^ Bioscience and Engineering Laboratory, FUJIFILM Corporation, Kaisei, Kanagawa, Japan.; ^2^ FUJIFILM Cellular Dynamics, Inc., Madison, Wisconsin.

**Keywords:** drug discovery, neurotherapeutic drugs, neuron, astrocyte

## Abstract

The strong barrier function of the blood–brain barrier (BBB) protects the central nervous system (CNS) from xenobiotic substances, while the expression of selective transporters controls the transportation of nutrients between the blood and brain. As a result, the delivery of drugs to the CNS and prediction of the ability of specific drugs to penetrate the BBB can be difficult. Although *in vivo* pharmacokinetic analysis using rodents is a commonly used method for predicting human BBB permeability, novel *in vitro* BBB models, such as Transwell models, have been developed recently. Induced pluripotent stem cells (iPSCs) have the potential to differentiate into various types of cells, and protocols for the differentiation of iPSCs to generate brain microvascular endothelial cells (BMECs) have been reported. The use of iPSCs makes it easy to scale-up iPSC-derived BMECs (iBMECs) and enables production of BBB disease models by using iPSCs from multiple donors with disease, which are advantageous properties compared with models that utilize primary BMECs (pBMECs). There has been little research on the value of iBMECs for predicting BBB permeability. This study focused on the similarity of iBMECs to pBMECs and investigated the ability of iPSC-BBB models (monoculture and coculture) to predict *in vivo* human BBB permeability using iBMECs. iBMECs express BMEC markers (e.g., VE-cadherin and claudin-5) and influx/efflux transporters (e.g., Glut-1, SLC7A5, CD220, P-gp, ABCG2, and MRP-1) and exhibit high barrier function (transendothelial electrical resistance, >1000 Ω × cm^2^) as well as similar transporter expression profiles to pBMECs. We determined that the efflux activity using P-glycoprotein (P-gp) transporter is not sufficient in iBMECs, while in drug permeability tests, iPSC-derived BBB models showed a higher correlation with *in vivo* human BBB permeability compared with a rat BBB model and the Caco-2 model. In a comparison between monoculture and coculture models, the coculture BBB model showed higher efflux activity for compounds with low CNS permeability (e.g., verapamil and thioridazine). In conclusion, iPSC-BBB models make it possible to predict BBB permeability, and employing coculturing can improve iPSC-BBB function.

## Introduction

Clinical trials of central nervous system (CNS) drugs have shown a very low overall success rate (6.2% vs. 13.3% for non-CNS drugs) and the time required for approval by the U.S. Food and Drug Administration (FDA) is longer (19.3 vs. 14.7 months for non-CNS drugs).^1^ The major reasons for failures in CNS drug development are (1) unknown drug distribution in the CNS and (2) a gap between pre-clinical and clinical data due to interspecies variation.^2^ One of the challenges in resolving these issues is the development of *in vitro* assays, as an alternative to animal models, which can accurately estimate pharmacokinetics in the CNS.^3^ *In silico* simulation models have been established to predict *in vivo* blood–brain barrier (BBB) permeability. The accuracy of *in silico* prediction of BBB permeability to small molecules (<1000 Da) has improved; however, it remains difficult to predict BBB permeability to noncovalent, inorganic, higher molecular weight, and mixtures of compounds using this model.^4^

The BBB is a key structure in the CNS for nutrients and drugs to penetrate from the blood vessels to the brain cortex, and the tight junctions (Tjs) formed by brain microvascular endothelial cells (BMECs) have a high barrier function (∼1800 Ω × cm^2^ *in vivo* in TEER: transendothelial electrical resistance) that protects the brain from neurotoxic substances.^5^ Conversely, the high barrier function of BMECs hinders the ability of neurotherapeutic drugs to exert therapeutic effects in the CNS. To investigate CNS pharmacokinetics, although animal models are frequently used, transporter expression in the BBB differs among animal species,^2^ which affects drugs penetration through the BBB. To date, in addition to animal models, Transwell permeability assays, such as the parallel artificial membrane permeability assay (PAMPA) and Caco-2 permeability assay, are used to predict *in vivo* BBB permeability; however, the predictive accuracy of these assays is insufficient due to use of non-BMEC cells in Transwell assays.^6^ In BBB models with primary BMECs (pBMEC) obtained from rat, bovine, or human brains, the predictive accuracy has been improved as a result^6–8^; however, obtaining pBMECs requires sacrificing a large number of animals and large-scale culture is an issue due to the limited yield of BMECs from donors. Recent studies have developed protocols for BMEC induction using stem cells such as embryonic stem (ES) cells, induced pluripotent stem cells (iPSCs), and hematopoetic stem cells (HPSCs) with high proliferation ability, which show properties similar, but not identical to pBMECs. Lippmann et al., reported protocols for differentiation of BMECs from ES cells and iPSCs, and iPSC-derived BMEC (iBMEC) showed high barrier function (>1000 Ω × cm^2^) as well as expression of EC markers, Tjs markers, glucose transporter 1 (Glut-1), and efflux transporters,^9,10^ and coculture of human iBMECs with pericytes, neurons, and astrocytes further increases the barrier function.^11^ Human HPSC-derived BMECs exhibit good predictive performance against human K_p,uu,CSF_, although the BBB barrier function (∼200 Ω × cm^2^)^12^ is not as high as that of the human BBB *in vivo.*^13^ Studies have compared the ability of *in vitro* and *in silico* models versus *in vivo* rodent models to predict BBB permeability to specific drugs^4,14–16^; however, few studies have succeeded in accurately predicting *in vivo* drug permeability in the human CNS.^12^ Therefore, in this study, we therefore investigated whether an *in vitro* BBB model using iBMECs can predict human *in vivo* drug permeability in the CNS.

## Materials and Methods

### Differentiation of BMECs from iPSCs

**FIG. 1.**
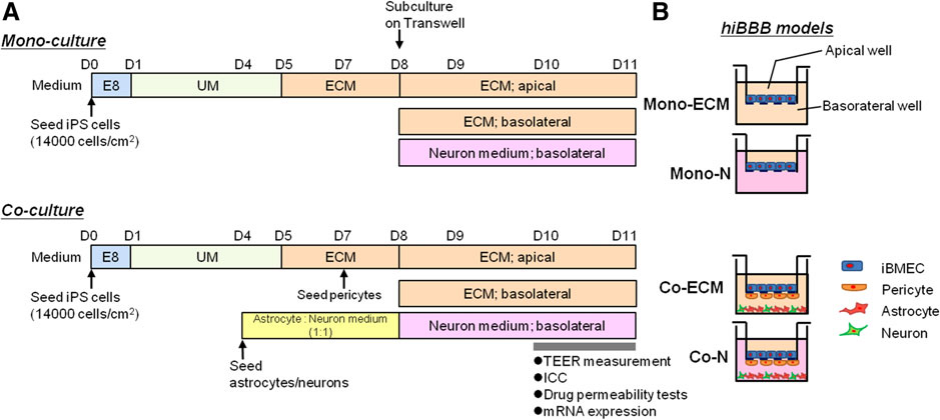
Schema of experimental protocols. **(A)** iPSCs were seeded on D0 and cultured in unconditional medium from D1–D5 and in ECM (+RA, +bFGF) from D5–D8 to differentiate BMECs. On D8, BMECs were subcultured on Transwells in ECM (+RA, +bFGF) on the apical side and the medium was changed to ECM (+RA) on D9. For monoculture, either ECM (+RA) or neuron medium was used in basolateral wells. For coculture, astrocytes and neurons were cultured in astrocyte/neuron medium (1:1) from D4–D8 and pericytes were seeded on the back side of the Transwell in pericyte medium on D7. BMECs were seeded on the upper side of the Transwells with pericytes and cultured with astrocytes/neurons on the basolateral side with either ECM or neuron medium from D8–D11. TEER was measured on D10 and D11, ICC was performed on D10, and drug permeability tests and mRNA were performed on D11. **(B)** Structures of hiBBB models. ECM is used in apical wells in all monoculture and coculture models. For Mono-ECM and Co-ECM, ECM was used in the basolateral wells, and for Mono-N and Co-N, neuron medium was used in the basolateral wells. bFGF, basic fibroblast growth factor; BMEC, brain microvascular endothelial cell; ECM, endothelial cell medium; hiBBB, human iPSC-derived blood–brain barrier; ICC, immunocytochemistry; iPSC, induced pluripotent stem cell; RA, retinoic acid; TEER, transendothelial electrical resistance.

iBMECs were differentiated from human iPSCs using differentiation protocols reported by Shusta and others^9,10,17,18^ with minor modifications. iPSCs (01279 line provided by FUJIFILM Cellular Dynamics, Inc. [FCDI], WI) were cultured on vitronectin-coated plates (1:100; Thermo Fisher Scientific, Inc., MA) with Essential 8 Flex Medium (E8; Gibco, MA) in an incubator at 37°C with 5% O_2_. To induce BMEC differentiation, 14,000 cells/cm^2^ of singularized iPSCs were seeded on a vitronectin-coated T150 flask in E8 with Y-27632 (10 μM; FUJIFILM Wako Pure Chemical Co. [Wako], Japan) on day 0 (D0). E8 was replaced with unconditioned medium (UM) containing 20% KnockOut Serum Replacement (Gibco), 1 × MEM nonessential amino acids (Gibco), 1 mM L-glutamine (Sigma-Aldrich, MO), and 0.1 mM β-mercaptoethanol (Wako) in DMEM/F12 (Gibco) on D1, and the UM was refreshed every day from D1–D4. Cells were subsequently cultured in human endothelial cell medium (ECM; Gibco) containing 1% platelet-poor plasma (PPP) serum (bovine derived; Thermo Fisher Scientific, Inc.; human-derived; Sigma-Aldrich), 20 ng/mL basic fibroblast growth factor (bFGF; R&D Systems, MN) and 10 μM retinoic acid (Sigma-Aldrich) from D5–D8, and the ECM was refreshed every other day. Cells were then harvested and seeded on Transwells (φ40 μm; Millipore, MA; or Transwell Permeable Supports for immunocytochemistry [ICC]; Corning, NY) coated with 0.1 mg/mL fibronectin (Wako) and 0.1 mg/mL Cellmatrix Type IV (Nitta Gelatin, Japan) at 1.0 × 10^6^ cells/cm^2^ in ECM, and the medium was replaced with ECM minus bFGF on D9. Cells were maintained in 5% O_2_ from D0–D8, and transferred to normoxic conditions from D8 after subculturing on Transwells. On D10 and D11, the induced BMECs were subjected to TEER measurement, ICC staining, mRNA extraction, and drug permeability assay (Fig. 1A).

### Coculture with astrocytes and neurons

For coculturing, astrocytes (0.12 × 10^6^ cells/cm^2^, iCell Astrocyte; FCDI) and neurons (0.04 × 10^6^ cells/cm^2^, iCell GABANeuron; FCDI) were cocultured on 24-well plates coated with 0.1 mg/mL poly-l-lysine (TREVIGEN, MD) and 3.3 μg/mL laminin-521 (BioLamina, Sweden) in 1:1 astrocyte/neuron medium purchased from FCDI on D4. On D8, 5.0 × 10^3^ cells/cm^2^ of primary human brain vascular pericytes (ScienCell, CA) were seeded on the basolateral side of Transwells by flipping the plate upside-down in a pericyte medium (ScienCell). A coculture BBB model was constructed as shown in Figure 1B. In our human iPSC-derived BBB (hiBBB) models, ECM or neuron medium was used in each monoculture BBB system (Mono-ECM and Mono-N) or coculture BBB system (Co-ECM and Co-N), respectively. In the apical wells, ECM was used for all groups (Fig. 1A, B).

### TEER measurement

TEER was measured on D10 and D11 using an EVOM2 voltohmeter with ENDOHM-6 chambers (World Precision Instruments, FL). The TEER was recorded at the peak value. Membrane resistance was calculated as TEER (Ω × cm^2^) = measured resistance value (Ω) × surface area (cm^2^).

### ICC staining

ICC was performed using an Image-iT Kit (Invitrogen, MA) on D10. Briefly, iBMECs on permeable Transwells were washed with Dulbecco's phosphate-buffered saline (DPBS) and fixed in 4% paraformaldehyde for 15 min at room temperature, followed by permeabilization with 0.5% Triton X-100 in DPBS for 10 min and blocking with Blocking Solution for 60 min. For staining, cells were incubated with primary antibodies (Table 1) overnight at 4°C, and subsequently stained with donkey anti-mouse IgG Alexa Fluor 488 or donkey anti-rabbit IgG Alexa Fluor 594 (1:1000; Thermo Fisher Scientific, Inc.) for 1 h at room temperature. Nuclei were stained with 4′,6-diamidino-2-phenylindole (DAPI, 1:10,000; Biotium, CA) for 15 min. Protein expression was visualized by fluorescence microscopy (BZ-X710; KEYENCE, Japan).

**Table 1.** Antibodies

Antibodies	Host	Dilution	Code no.	Supplier
VE-cadherin	Rabbit	1:50	ab33168	Abcam, UK
vWF	Rabbit	1:50	ab6994	Abcam
Ulex		1:50	FL-1061-2	Vector Laboratories, Inc., CA
Glut-1	Mouse	1:50	MS-10637-P0	Thermo Fisher Scientific, Inc.
CD220	Mouse	1:50	MA5-13778	Invitrogen
MRP-1	Mouse	1:25	MAB4155	Millipore
P-gp	Rabbit	1:50	ab170904	Abcam
SLC7A5	Rabbit	1:100	HPA052673	Atlas Antibodies, Sweden
ABCG2	Mouse	1:50	MAB4100	Millipore
Occludin	Mouse	1:50	33–1500	Invitrogen
Claludin-5	Mouse	1:50	35–2500	Invitrogen
ZO-1	Rabbit	1:50	61–7300	Invitrogen

### Caco-2 and ratBBB kits

To compare drug permeability with non-BMEC and non-human cells, we performed drug permeability tests using Caco-2 human epithelial colorectal adenocarcinoma cells and rat pBMECs. A Caco-2 kit (KAC Co. Ltd., Japan) was used according to the manufacturer's instructions. Briefly, the medium was refreshed and the kits were incubated overnight and used for permeability tests the next day.

RatBBB kits (RBE-12; PharmaCo-Cell Company Ltd., Japan) were maintained according to the manufacturer's instructions. Briefly, the kits were thawed by adding thawing solution and subsequently cultured with Culture medium 1 (500 μL in apical well and 1500 μL in basolateral well). After overnight incubation, the medium was replaced with Culture medium 2 and the cells were maintained for 4 days before use in drug permeability tests. Both drug permeability tests using Caco-2 and ratBBB kits were performed following the protocol described for drug permeability tests in the Material and Methods section.

### Drug transporter array

All reagents and array plates were purchased from Qiagen-SABioscience. Cells were extracted with lysis buffer on D11, homogenized with a QIAshredder, and mRNA was purified using an RNeasyPlus Micro Kit with DNaseI. Transcription of cDNA from mRNA was performed using an RT^2^ First Strand Kit, and human drug transporter array (RT^2^ Profiler PCR Array Human Drug Transporters, 330231 PAHS-070Z) was performed with RT^2^ SYBR Green qPCR Mastermixes. All procedures were performed according to the manufacturer's instructions. Real-time polymerase chain reaction (PCR) was performed on a ViiA 7 Real-Time PCR System, and data analysis was performed using R statistical software (version 3.5.2). Heat map data were drawn based on ΔCt values normalized to the average of eight housekeeping genes (i.e., *Tap1, Tap2, Vdac1, Vdac2, B2m, GAPDH, Hprt1,* and *RPLPO*) and Ward's clustering method was used for the analysis.

### Drug permeability tests

Cells were incubated with Hank's Balanced Salt Solution (HBSS) (Wako) for 2 h on D11 and the TEER was measured before carrying out permeability tests. hiBBB models with barrier function less than 500 Ω × cm^2^ were excluded from drug permeability tests. For Caco-2 and ratBBB kits, all Transwells were used for permeability tests. Drugs at a concentration of 10 μM (Tables 2 and 3) diluted in HBSS were added to apical wells for A-to-B samples or basolateral wells for B-to-A samples; the cells were then incubated at 37°C with agitation and 50-μL samples from the basolateral wells (A-to-B samples) and apical wells (B-to-A samples) were collected at 60 and 80 min. Drug concentrations were measured by LC/MS analysis (LC: Prominence, MS: LCMS-2010EV; Shimazu Co., Japan), and LC/MSMS analysis (LC: Acquity; Waters Co., MA; MS: TSQ QUANTIVA; Thermo Fisher Scientific, Inc.) was used for digoxin measurement. For the data analyses, Papp (apparent permeability coefficients; cm/sec), and efflux ratios were calculated using the following formulas:

\begin{align*}
{\rm Papp}{ \rm{ }} \left(\hbox{cm / sec} \right) \; = \;\hbox{dQ / dT} \; \times \;{\rm A} \; \times \;{\rm C}0
\end{align*}

\begin{align*}
{\rm Efflux}{ \rm{ }} \ {\rm ratio} \; = \;{\rm Papp}{ \rm{ }} \left( {{\rm B} - {\rm to} - {\rm A}} \right) / {\rm Papp}{ \rm{ }} \left( {{\rm A} - {\rm to} - {\rm B}} \right)
\end{align*}

where dQ/dT is the amount of drug transported per unit time; A is the membrane surface area; and C0 is the donor concentration at time 0.

**Table 2.** Drug Permeability test 1: P-Glycoprotein Substrates

Drugs	MW	Solvent	Code no.	Suppliers
Digoxin	780.9	DMSO	B21902	Alfa Aesar, UK
Colchicine	399.4	DMSO	039-03851	Wako
Quinidine	324.4	DMSO	176-00111	Wako
Vinblastine	909.1	DMSO	11762	Cayman Chemical, MI
Glibenclamide	494.0	DMSO	078-03881	Wako
Caffeine (simple diffusion)	194.2	DMSO	C0750	Sigma-Aldrich

DMSO, dimethyl sulfoxide; MW, molecular weight.

**Table 3.** Drug Permeability Test 2: Comparison with Human *In Vivo* Permeability

Drugs	MW	Solvent	Code no.	Suppliers	CSF/plasma in human^27^
Bupropion	276.2	H_2_O	028-17311	Wako	0.43
Gabapentin	171.2	H_2_O	076-05641	Wako	0.113
Lamotrigine	255.0	DMSO	L0349	LKT Laboratories, Inc., MI	0.43
Tacrine	234.7	DMSO	70240	Cayman Chemical	0.39
Thioridazine	407.0	DMSO	T9025	Sigma-Aldrich	0.01
Topiramate	339.4	DMSO	T540250	Toronto Research Chemicals, Canada	0.84
Verapamil	454.6	H_2_O	V4629	Sigma-Aldrich	0.068

CSF, cerebrospinal fluid.

### Statistics

Comparisons of more than three groups were performed using two-way analysis of variance (ANOVA) followed by Tukey's–Kramer test. For TEER measurement, two-way repeated measures ANOVA followed by Bonferroni–Dunn test was used. Statistical significance was expressed as *p < 0.05 and **p < 0.01. Statcel3 software (OMS Publishing, Inc., Japan) was used for all statistical analyses. All values are presented as mean ± standard deviation, unless otherwise indicated.

## Results

### Barrier function

**FIG. 2.**
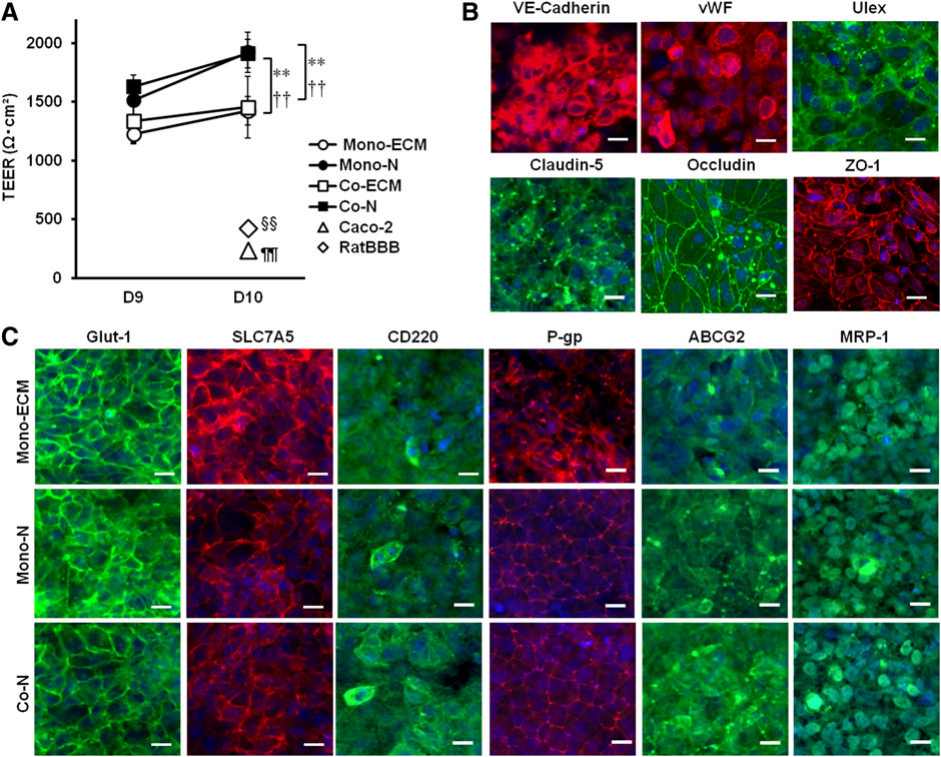
TEER and ICC. **(A)** All differentiated BMEC groups had high barrier function (>1000 Ω × cm^2^), while Caco-2 and ratBBB kits showed barrier function <500 Ω × cm^2^. Both Mono-N and Co-N showed higher TEER values compared with the other groups. **p < 0.01, versus Mono-ECM; ^††^*p* < 0.01, versus Co-ECM; ^§§^*p* < 0.01, RatBBB versus the other groups; Caco-2 versus the other groups. The error bars show the SEM. **(B)** ICC staining of endothelial cell marker (VE-cadherin, vWF, and Ulex) and tight junction marker (claudin-5, occludin, and ZO-1). iBMECs expressed endothelial cell markers and tight junction proteins. **(C)** Expression of transporter proteins in iBMECs. Glucose transporter 1 (Glut-1), solute carrier family 7 member 5 (SLC7A5), CD220 (insulin receptor), P-glycoprotein (P-gp), ATP binding cassette subfamily G member 2 (ABCG2), and multidrug resistance-associated protein 1 (MRP-1) were expressed in BMECs and all groups showed almost the same degree of expression. Nuclei were visualized with DAPI (Blue). Scale bars = 20 μm. DAPI, 4′,6-diamidino-2-phenylindole; iBMEC, iPSC-derived BMEC; SEM, standard error of the mean.

We initially compared the effects of PPP in the Mono-ECM model between bovine (bPPP) and human derived (hPPP). hPPP remarkably improved the differentiation efficiency compared with bPPP (100% in hPPP vs. 66.7% in bPPP), and moreover, significantly increased the barrier function compared with the use of bPPP on D9 (hPPP, 1223 ± 406, *n* = 24, vs. bPPP, 919 ± 306, *n* = 48, Ω × cm^2^, *p* < 0*.05*); we therefore used hPPP in subsequent ECM model experiments. All hiBBB models showed remarkably high barrier function >1000 Ω × cm^2^. Neuron medium in basolateral wells significantly increased the TEER compared with ECM in both monoculture and coculture models on D10 (Mono-N, 1920 ± 774, *n* = 20; Co-N, 1908 ± 582, *n* = 23 vs. Mono-ECM, 1423 ± 592, *n* = 24; Co-ECM, 1454 ± 263, *n* = 7; Ω × cm^2^). Caco-2 and ratBBB showed significantly lower TEER values compared with iBMECs (Caco-2, 239 ± 97, *n* = 20; ratBBB, 425 ± 67, *n* = 24, on D10; Ω × cm^2^). Taken together, these results suggest the neuron medium greatly improved the barrier function of the hiBBB model (Fig. 2A).

### BMEC markers in iBMECs

iBMECs, differentiated according to the protocol employed, expressed EC markers (i.e., VE-cadherin, vWF, and Ulex), Tjs proteins (i.e., claudin-5, occluding and ZO-1) (Fig. 2B). All groups showed similar expression of influx transporters (i.e., Glut-1, SLC7A5, and CD220) and efflux transporters (i.e., P-pg, ABCG2 and MRP-1)^10^ (Fig. 2C). These results demonstrated that both monoculture with neuron medium in basolateral wells and coculture with pericytes, astrocytes, and neurons did not affect the expression of BMEC markers.

### Drug transporter expression

**FIG. 3.**
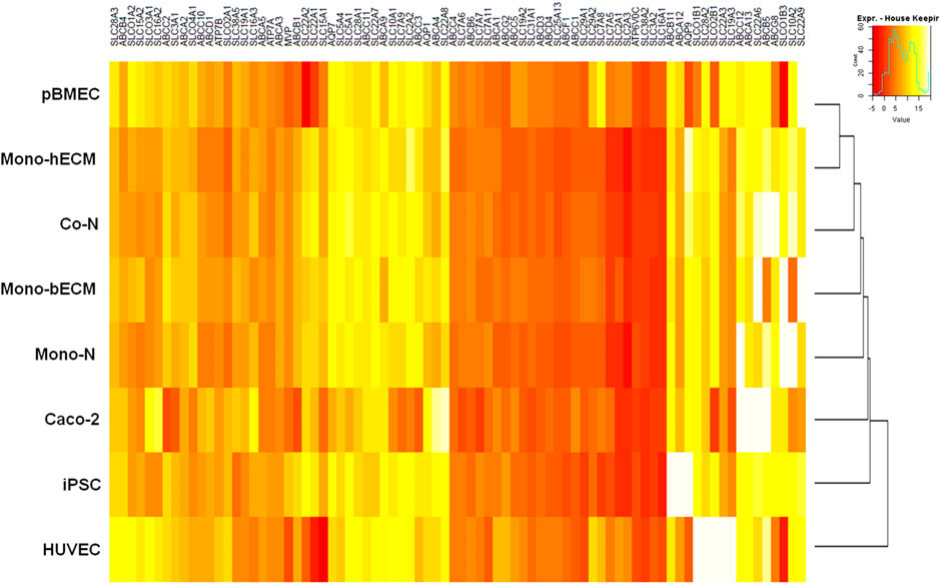
Drug transporter array heat map. Mono-hECM showed the highest similarity to the transporter expression profiles of pBMECs. All hiBBB models showed similar expression profiles to pBMEC. Caco-2 cells, iPSCs, and HUVECs had expression profiles that differed between the pBEMC and hiBBB models. Data are shown as ΔCt. HUVEC, human umbilical cord blood cell; pBMEC, primary BMEC.

Transporter expression profiles of iBMECs (*n* = 3) were compared with pBMECs (*n* = 1), Caco-2 cells (*n* = 1), iPSCs (*n* = 2), and human umbilical cord blood cells (HUVECs, *n* = 1). In contrast to HUVECs, iPSCs, and Caco-2 cells, the hiBBB models showed very similar expression profiles to that of pBMECs. In comparison to hPPP (Mono-hECM) and bPPP (Mono-bECM), pBMEC showed a closer expression profile to Mono-hECM than to Mono-bECM. There was almost no difference in transporter expression among the hiBBB models. With respect to typical efflux transporters, Caco-2 cells clearly showed higher expression of *P-gp/ABCB1* versus other cell types, while *ABCC1/BCRP* expression was almost the same among pBMECs, hiBBB models, and Caco-2 cells, and *ABCG2/MRP-1* expression was the same between pBMEC and hiBBB models, but lower in Caco-2 cells (Fig. 3).

### Drug permeability tests

We investigated drug permeability of Co-N, which most closely mimics the *in vivo* properties of the BBB and has frequently been used as an *in vitro* BBB model^2,10^ comparing Mono-bECM, reported by Lippmann et al. as the original iBMECs,^9^ comparing Caco-2 and ratBBB kits. We first tested the drugs listed under permeability test 1 of Table 2, which have been reported to be P-glycoprotein (P-gp) substrates.^19–21^ Caffeine was used as a positive control for simple diffusion across the BBB and it showed high permeability in all *in vitro* BBB models. Mono-ECM showed a remarkably high efflux ratio in vinblastine and digoxin, and showed <2.0 efflux ratio in quinidine, colchicine, and glibenclamide.

**FIG. 4.**
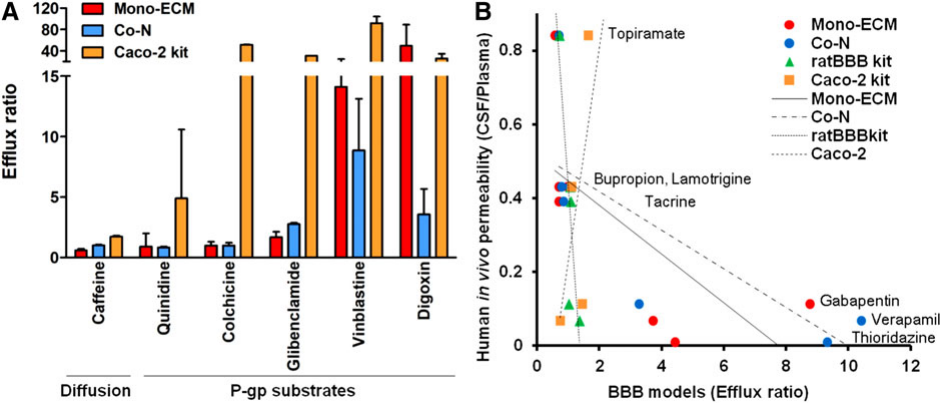
Drug permeability tests. **(A)** Permeability of P-gp substrates. Simple diffusion compound: caffeine showed high permeability in all groups. Mono-ECM and Co-N showed high efflux activity with vinblastine and digoxin, and low efflux activity with quinidine, colchicine, and glibenclamide. Caco-2 showed high efflux activity for all P-gp substrates. Error bars show SEM. **(B)** Prediction of human *in vivo* drug permeability. For topiramate, bupropion, lamotrigine, and tacrine, all BBB models showed similar drug permeability. Mono-ECM and Co-N models showed high efflux activities for gabapentin, verapamil, and thioridazine and had a stronger correlation with human *in vivo* permeability. RatBBB and Caco-2 kits had low efflux ratios with gabapentin and verapamil, which have low central nervous system permeability, and correlated poorly with human *in vivo* data.

Co-N showed >2.0 high efflux ratio in glibenclamide, vinblastine, and digoxin, and showed <2.0 efflux ratio in quinidine and colchicine. Caco-2 cells showed high efflux activity for all P-gp substrates (Fig. 4A). We subsequently investigated the correlation between *in vivo* human drug permeability (Table 3, permeability test 2) and drug permeability of iBMECs (Mono-ECM and Co-N) compared with ratBBB and Caco-2 kits. hiBBB models showed a greater correlation with *in vivo* data compared with Caco-2 kit, with an efflux ratio greater than two for gabapentin, verapamil, and thioridazine, which are reported to have low CNS permeability (Mono-ECM, *R*^2^ = 0.49; Co-N, *R*^2^ = 0.60; Caco-2 kit, *R*^2^ = 0.41). RatBBB kit showed higher correlation than hiBBB (ratBBB kit, *R*^2^ = 0.73); however, high concentration of verapamil and gabapentin penetrated to basolateral well across ratBMECs, which indicates that the ratBBB kit is inappropriate as a drug permeability prediction tool in substrates for efflux transporter. Taken together, these results suggest that P-gp transporter expression is insufficient in hiBBB models; however, hiBBB models have a better ability to predict drug permeability compared with other prediction models.

## Discussion

Although some reports have indicated benefits of using iBMECs for *in vitro* BBB modeling,^12,17^ the degree of similarity between iBMECs and pBMECs is not well known. We therefore focused on the ability of hiBBB models to predict *in vivo* drug permeability, and the transporter expression profiles of iBMECs compared with pBMECs. We first confirmed that iBMECs express BMEC markers (i.e., Tjs, EC marker, and influx/efflux transporters). In addition, this study demonstrated that neuron medium improves barrier function without coculturing, and that hPPP improves the reproducibility of iBMEC differentiation. Given that PPP contains angiogenic factors such as vascular endothelial growth factor, bFGF, platelet-derived growth factor, angiopoietin, and epidermal growth factor, the robustness of BMEC differentiation might be affected by PPP's angiogenic potential,^22^ as suggested by the difference between hPPP and bPPP. Indeed, in drug transporter array, pBMEC had a closer expression profile to Mono-hECM than to Mono-bECM, which indicates that the formulation of PPP is crucial for controlling BMEC function. Using the method of Lippoman et al., we succeeded in shortening the differentiation period and improving the robustness of BMEC differentiation.

Transporter expression profiles in iBMECs were similar to pBMECs, while the expression of some transporters was remarkably higher or lower in iBMECs. Higher expression in iBMEC was mostly observed for the SLC2 family of glucose transporters (i.e., *SLC2A1* and *SLC2A3*) and the SLC22 family, which transports organic anions/cations (i.e., *SLC22A3* and *SLC22A8*)^23^; however, there was no consistent pattern. P-gp on BMECs is a gatekeeper protein for xenobiotics^24^ and serves as an essential transporter to protect the CNS; however, *P-gp* expression was low in iBMECs, even though ICC clearly demonstrated P-gp expression. In iBMECs, the insufficient expression of *P-gp* is one of the features that needs to be improved upon to accurately predict the permeability of P-gp substrates. Indeed, hiBBB models (e.g., Mono-ECM and Co-N) fail to exert efflux activity with some P-gp substrates such as quinidine, colchicine, and glibenclamid.^19,21,25^ In contrast, these models showed sufficiently high efflux activity in digoxin, verapamil, and vinblastine, which are known to be P-gp substrates. Since the transporter expression profiles of *in vivo* BMECs remain unclear,^26^ it is important to more thoroughly understand the transporter expression of *in vivo* BMECs, and manipulating the transporter expression of iBMECs to be comparable to that of *in vivo* BMECs is crucial for developing *in vivo*-like hiBBB models.

To assess the ability of *in vitro* BBB models to predict drug permeability, we selected drugs for which *in vivo* pharmacodynamics in the human CNS have been previously established.^27^ Both ratBBB and Caco-2 kits showed poor predictive ability, while hiBBB models (both monoculture and coculture) correlated with human *in vivo* permeability. Drugs with high BBB permeability, such as topiramate, bupropion, lamotrigine, and tacrine, showed the same permeation ratios among the groups tested. Although transporters for topiramate, bupropion, lamotrigine, and tacrine have been identified as P-gp, dopamine transporter,^28^ organic cation transporter 1 (OCT1),^29^ and choline transporter,^30^ respectively, not all transporters for these drugs have been determined. In this study, their high distribution in the CSF, as shown in *in vivo* studies,^27^ was confirmed by the high permeability observed in the hiBBB models. Gabapentin is a substrate for SLC7A5 influx transporter,^31^ which is known to localize at the BBB; however, based on *in vivo* studies, it shows poor distribution in the CNS.^32^ The drug transporter array showed, in iBMECs, the extremely high *SLC7A5* expression, while the permeability of gabapentin was poor in the hiBBB model employing iBMECs. Thus, transporter expression and *in vivo* permeability do not necessarily correspond. Verapamil is widely known as an efflux compound by P-gp,^33^ and hiBBB models exhibited efflux activity for verapamil, while both ratBBB kit and Caco-2 kits showed low efflux activity for verapamil. Taken together, these results clearly indicate that drug permeability in hiBBB models connote mechanisms implicating efflux transport of gabapentin, verapamil, and thioridazine.

## Conclusion

The hiBBB model is more reliable for predicting drug permeability compared with non-human and non-BMEC BBB models. Further elucidation of transport mechanisms by the BBB is essential for improving predictive accuracy.

## Abbreviations Used

ABCG2: ATP binding cassette subfamily G member 2
ANOVA: analysis of variance
BBB: blood–brain barrier
bFGF: basic fibroblast growth factor
BMEC: brain microvascular endothelial cell
CNS: central nervous system
CSF: cerebrospinal fluid
DAPI: 4′,6-diamidino-2-phenylindole
DMSO: dimethyl sulfoxide
DPBS: Dulbecco's phosphate-buffered saline
ECM: endothelial cell medium
ES cell: embryonic stem cell
Glut-1: glucose transporter 1
HBSS: Hank's Balanced Salt Solution
hiBBB: human iPSC-derived BBB
HPSCs: hematopoietic stem cells
HUVEC: human umbilical cord blood cell
iBMEC: iPSC-derived BMEC
ICC: immunocytochemistry
iPSCs: induced pluripotent stem cells
LC/MS: liquid chromatography–mass spectrometry
LC/MSMS: liquid chromatography-tandem mass spectrometry
MRP-1: multidrug resistance-associated protein 1
MW: molecular weight
OCT1: organic cation transporter 1
PAMPA: parallel artificial membrane permeability assay
pBMEC: primary BMEC
P-gp: P-glycoprotein
PPP: platelet poor plasma
RA: ratinoic acid
SEM: standard error of the mean
SLC7A5: solute carrier family 7 member 5
TEER: transendothelial electrical resistance
Tjs: tight junctions
Ulex: Ulex europaeus
UM: unconditioned medium
vWF: Von Willebrand factor
